# Supplementary material for: Primary Amoebic Meningoencephalitis caused by Complement C2 Deficiency
Source: medRxiv. 2026 Feb 2:2026.01.31.26345168. Preprint. [Version 1] doi: 10.64898/2026.01.31.26345168 (PMC12889776; doi:10.64898/2026.01.31.26345168)
Supplement: Supplement 1 [file media-1.pdf]

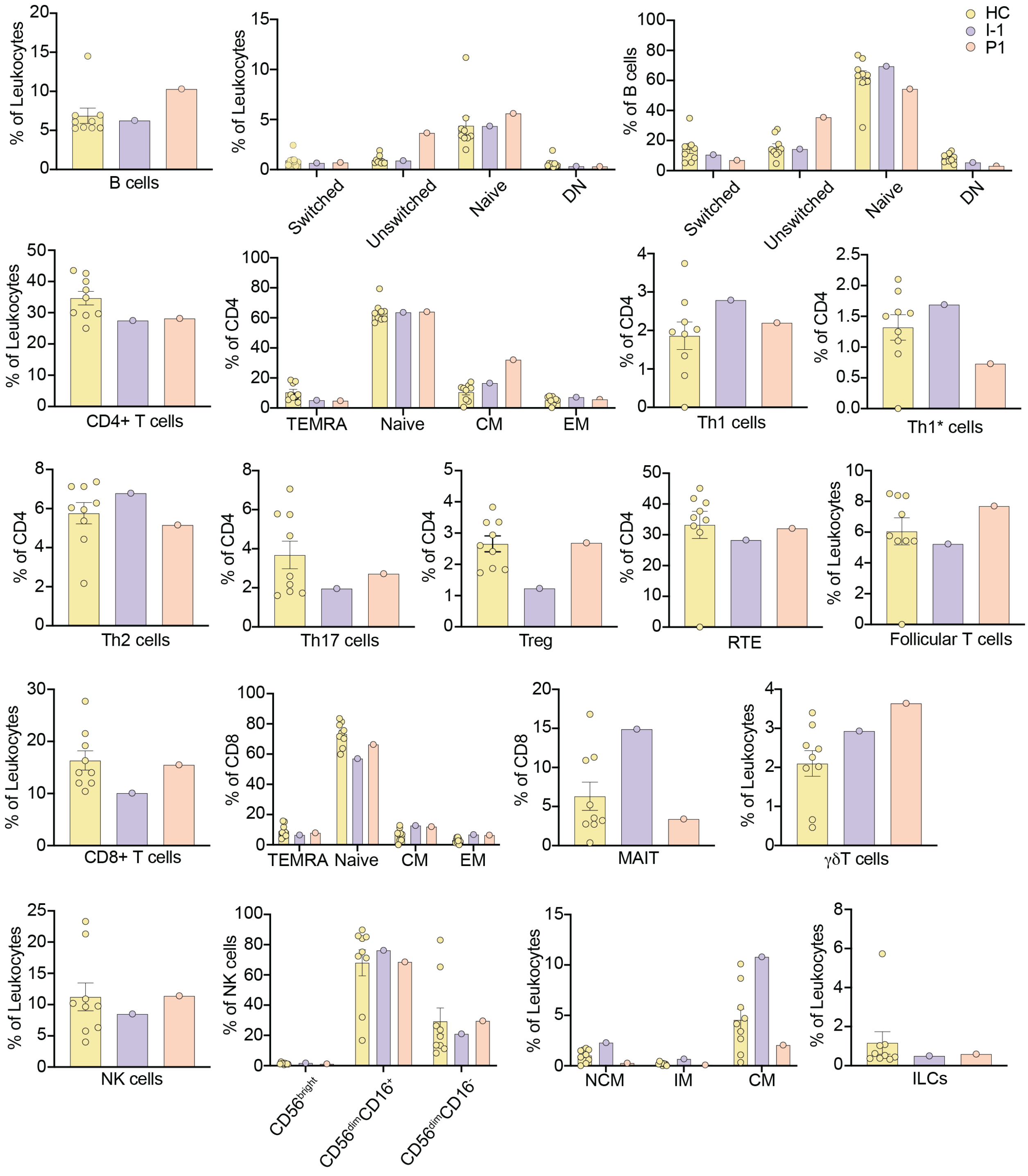

Supplementary Figure S1. Immunophenotyping frequencies by manual gating. Frequencies of immune populations as a percentage of leukocytes or parent populations in healthy controls (HC), parent of the patient (I-1), and the patient (P1)

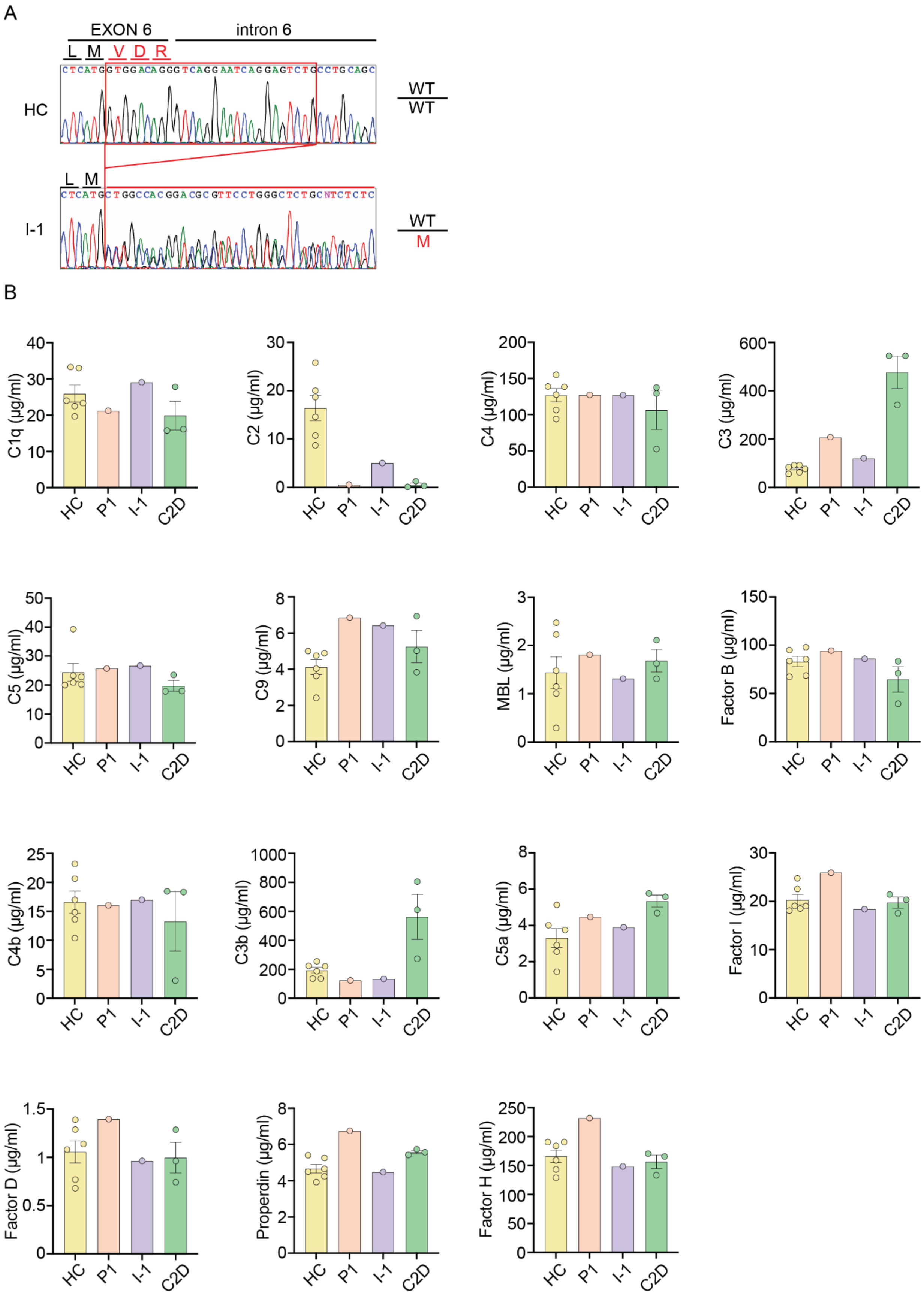
A
EXON 6
intron 6
L M V D R
HC
WT
WT
L M
I-1
WT
M
B
C1q (µg/ml)
C2 (µg/ml)
C4 (µg/ml)
C3 (µg/ml)
C5 (µg/ml)
C9 (µg/ml)
MBL (µg/ml)
Factor B (µg/ml)
C4b (µg/ml)
C3b (µg/ml)
C5a (µg/ml)
Factor I (µg/ml)
Factor D (µg/ml)
Properdin (µg/ml)
Factor H (µg/ml)
HC
P1
I-1
C2D

Supplementary Figure S2: Genetic characterization and complement component level quantification.

(A) Sanger sequencing showed a heterozygous mutation in C2 in a parent of the patient (I-1) compared to a healthy control (HC).

(B) Serum levels of complement components and associated factors measured in the patient (P1), a parent of the patient (I-1), and unrelated individuals with C2D, compared to healthy controls (HC).

Each bar represents mean ± SEM; individual data points are shown.

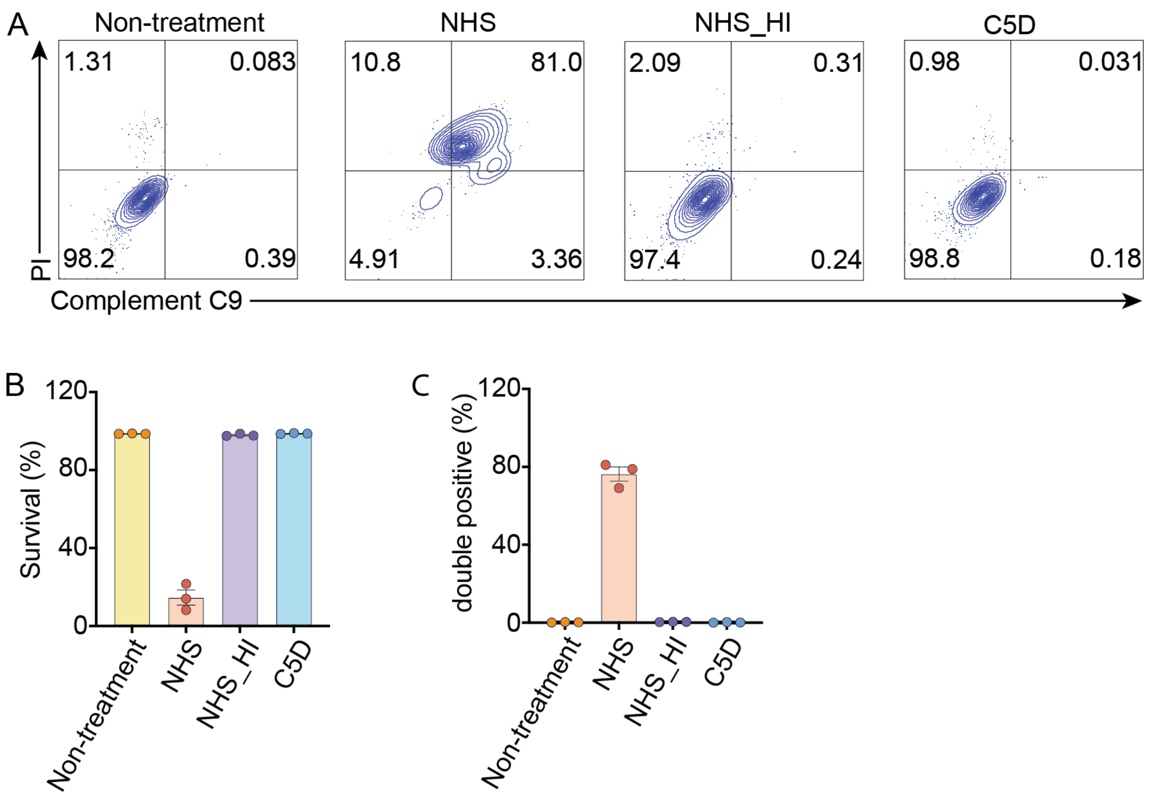

Supplementary Figure S3: Complement-Mediated Cytotoxicity of *N. fowleri*.

(A) Flow cytometry analysis of *N. fowleri* trophozoites following treatment with normal human serum (NHS), heat-inactivated NHS (NHS_HI), or C5-depleted serum (C5D). Cells were stained for complement C9 deposition (x-axis) and necrotic cell death using PI (y-axis).

(B) Quantification of trophozoite survival based on exclusion of PI staining.

(C) Percentage of double-positive cells (C9+ PI+) indicating complement-mediated lysis.

Data represent three independent experiments, mean ± SEM from biological replicates.

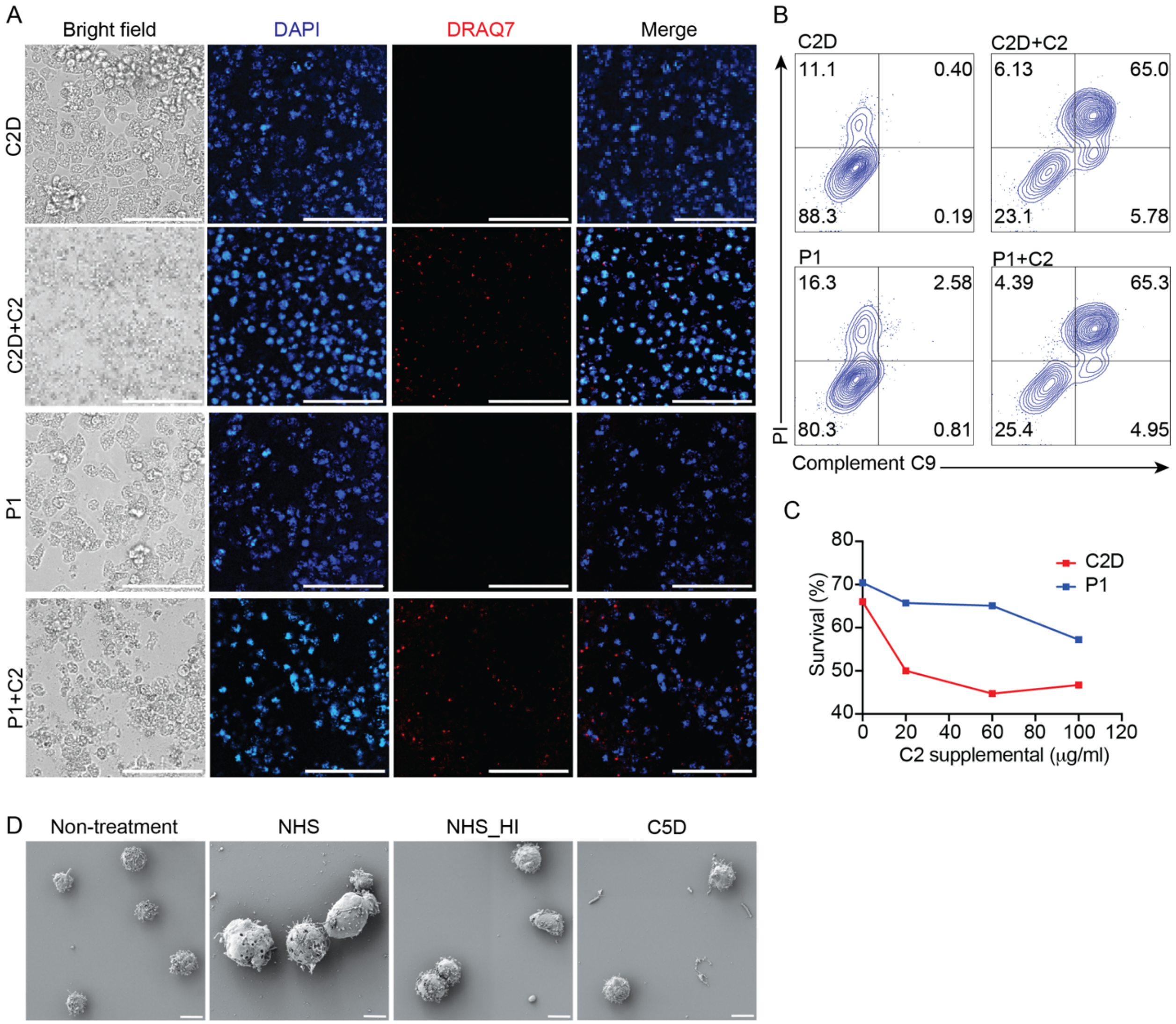

Supplementary Figure S4: C2 Supplementation Restores Complement-Mediated Killing of *N. fowleri* by C2-Deficient Serum.

(A) Immunofluorescence microscopy of *N. fowleri* incubated with C2-depleted serum (C2D), serum from the C2-deficient patient (P1), or the same sera supplemented with purified human C2 (C2D+C2, P1+C2). Cells were stained with DAPI (blue, nuclei) and DRAQ7 (red, necrotic/dead cells). Scale bar: 100 μm.

(B) Flow cytometric analysis of complement C9 deposition (x-axis) and cell death (PI, y-axis) on *N. fowleri* treated with the same serum conditions as in A.

(C) Dose-response curve for survival of *N. fowleri* in C2-deficient sera supplemented with increasing concentrations of purified C2.

(D) Electron microscopy of *N. fowleri* with non-treatment or treated with NHS, NHS-HI, and C5D. Scale bar: 10 µm. NHS: normal human serum; NHS-HI: normal human serum heat inactivation; C5D: C5 depleted serum

Data represent three independent experiments, mean ± SEM from biological replicates.

Supplementary Table S1: Antibodies used for CyTOF

Antibody	Clone	Isotype	Dilution	Company
Anti-Human CD45	H130	89Y	1:50	Standard BioTools
Anti-Human CD19	HIB19	142Nd	1:50	Standard BioTools
Anti-Human CD127/IL7Ra	A019D5	143Nd	1:50	Standard BioTools
Anti-Human CD38	HIT2	144Nd	1:50	Standard BioTools
Anti-Human IgD	IA6-2	146Nd	1:50	Standard BioTools
Anti-Human CD11c	Bu15	147Sm	1:50	Standard BioTools
Anti-Human CD16	3G8	148Nd	1:50	Standard BioTools
Anti-Human CD194/CCR4	L291H4	149Sm	1:50	Standard BioTools
Anti-Human CD123/IL-3R	6H6	151Eu	1:50	Standard BioTools
Anti-Human TCRγδ	11F2	152Sm	1:50	Standard BioTools
Anti-Human CD185/CXCR5	RF8B2	153Eu	1:50	Standard BioTools
Anti-Human CD3	UCHT1	154Sm	1:50	Standard BioTools
Anti-Human CD45RA	HI100	155Gd	1:50	Standard BioTools
Anti-Human CD27	L128	158Gd	1:50	Standard BioTools

Anti-Human CD28	CD28.2	160Gd	1:50	Standard BioTools
Anti-Human CD66b	80H3	162Dy	1:50	Standard BioTools
Anti-Human CD183/CXCR3	G025H7	163Dy	1:50	Standard BioTools
Anti-Human CD161	HP-3G10	164Dy	1:50	Standard BioTools
Anti-Human CD45RO	UCHL1	165Ho	1:50	Standard BioTools
Anti-Human CD24	ML5	166Er	1:50	Standard BioTools
Anti-Human CD197/CCR7	G043H7	167Er	1:50	Standard BioTools
Anti-Human CD8	SK1	168Er	1:50	Standard BioTools
Anti-Human CD25	2A3	169Tm	1:50	Standard BioTools
Anti-Human CD20	2H7	171Yb	1:50	Standard BioTools
Anti-Human HLA-DR	L243	173Yb	1:50	Standard BioTools
Anti-Human CD4	SK3	174Yb	1:50	Standard BioTools
Anti-Human CD56	NCAM16.2	176Yb	1:50	Standard BioTools
Anti-Human CD31	WM59	145Nd	1:50	Standard BioTools
Anti-Human CD196	G034E3	141Pr	1:25	Standard BioTools

Anti-Human CD14	M5E2	175Lu	1:25	Standard BioTools
Anti-Human CD117	104D2	150Nd	1:25	Biolegend
Anti-Human TCR Vα24/Jα18	6B11	156Gb	1:25	Biolegend
Anti-Human TCR Vα7.2	3C10	159Tb	1:25	Biolegend
Anti-Human CD294	BM16	161Dy	1:25	Biolegend

Supplementary Table S2: primers used for Sanger sequencing

Forward primer	5' – AAA GCC TGG GCC GTA AAA TCC – 3'
Reverse primer	5' – GAA GAC TTC TTG GAG GAG GTG GG – 3'

Supplementary Table S3: primers used for *N. fowleri* PCR test

Forward primer (NFITSFW)	5' – TGA AAA CCT TTT TTC CAT TTA CA – 3'
Reverse primer (NFITSRV)	5' – AAT AAA AGA TTG ACC ATT TGA AA – 3'
Forward primer (JITSFW)	5' – GTC TTC GTA GGT GAA CCT GC – 3'
Reverse primer (JITSRV)	5' – CCG CTT ACT GAT ATG CTT AA – 3'
